# Intergenerational Programs Involving Adolescents, Institutionalized Elderly, and Older Volunteers: Results from a Pilot Research-Action in Italy

**DOI:** 10.1155/2018/4360305

**Published:** 2018-12-05

**Authors:** Sara Santini, Valentina Tombolesi, Barbara Baschiera, Giovanni Lamura

**Affiliations:** ^1^IRCCS INRCA-National Institute of Health and Science on Ageing, Centre for Socio-Economic Research on Ageing, Ancona, Italy; ^2^IRCCS INRCA-National Institute of Health and Science on Ageing, Centre for Welfare Models and New Technologies, Ancona, Italy; ^3^Ca' Foscari University, Venice, Italy

## Abstract

Changes in traditional family structures, public policy arrangements, and new family care patterns are reducing opportunities for interaction between younger and older people in Europe and in Italy, especially when the latter live in residential care facilities. This may bolster age-related stereotypes in both generations and end up with affecting older people's self-confidence, devaluing their emotional and relational capabilities. “*Let's Re-Generate*” is a pilot research-action project—based on an intergenerational program carried out in a nursing home in Central Italy—which aimed at prompting institutionalized older people and adolescents in the community to enhance intergenerational ties through various shared activities. Results from focus groups with 25 teenagers and in-depth interviews with 16 older residents and 16 older volunteers provide an in-depth insight on the positive impact of intergenerational programs, which foster the interaction between different ages, help overcome age-related stereotypes, and improve older people's mental well-being and older volunteers' generativity. The main recommendation emerging from this study is that intergenerational activities should be integrated in the daily routine of nursing homes, acting as useful tools for fostering older residents' capability of reacting to dependency and social isolation.

## 1. Introduction

The nature of intergenerational relationships and their evolution over time may be influenced by different factors. Recent changes in these relationships have been explained by different theories on the evolution of family patterns [1–4], by macrolevel analyses of public policy arrangements [5], and by the study of environmental, economic, and social factors, such as the level of urbanization and the wealth of local communities [6, 7]. According to the latter, being in contact and having a relationship with a person of another age is not only a matter of personal choice, but it also depends on the chances of meeting and interacting with people in a social context [8].

The quantity and quality of intergenerational relationships also affect the capacity of transferring support and resources across generations, with a crucial impact on welfare state policies [9]. For example, weakening of intergenerational ties might reduce the propensity of adults and youngsters to take care of older parents and relatives once these become no longer independent, thus posing a substantial societal and policy challenge in light of the progressive population ageing taking place in Europe and worldwide [10].

With regard to the Italian context, the country on which this article is focused, a trend has been observed in the attitude of an increasing number of Italians, especially “Millennials” (i.e., the people born in the 1980s or 1990s), who are refusing to have relationships with people of another age in different contexts (e.g., workplace, vacation, and health services) [11].

Moreover, in this country intergenerational solidarity has been pursued by means of privately employed migrant care workers (MCW), who are increasingly substituting relatives in providing daily tailored hands-on care to older recipients [12–15].

A minority of Italian households, corresponding to no more than 2% of the over 65-year-old population (*Istituto Nazionale di Statistica *[ISTAT], National Institute of Statistics [16]), resorts to residential facilities for their older family members, which usually host older people with high levels of disability and without family network support and thus provide mainly medical and nursing care. These do not, however, provide sufficient psychosocial educational activities leaving most of the residents without motivating instances [17]. The only interpersonal relationships of the nursing home residents are often those with the healthcare professionals, whereas the contacts with the outside world, e.g., with volunteers, are very rare [17], causing older people to experience social isolation and emotional impoverishment [18–20]. Institutionalized older people, therefore, can be considered among the most excluded social groups in Italy because when they enter the residential facility, every interaction which could lead to nurturing thoughts and behaviours aimed at the intergenerational solidarity is hindered.

Another signal of a possible “intergenerational crumbling” [21] in the Italian context comes from the observation that part of the older population falls victim to abuse (physical, psychological, and financial) or neglect at the hand of relatives, children, and grandchildren in the first place, especially when older people live alone or are institutionalized [22].

The literature shows that frequent and meaningful direct contacts between cohorts of different generations can help overcome ageism, improve attitudes towards the other generation, and create intergenerational solidarity [7]. Thus, also in light of the abovementioned trends, it might also be appropriate to promote interventions based on the interaction between different generations, especially in contexts where older people are at risk of reduced meaningful relationships, e.g., in nursing homes. One of the main tools to systematically address the issue identified above is represented by the so-called intergenerational programs (IGPs), of which two main types are identified in the literature.

The first type embeds community-based programs involving community-dwelling older adults, which are mostly carried out in public social centres [23–25] and schools [26–28]. This type includes intergenerational service–learning [29–32] and reverse mentoring programs [33, 34], aimed at enhancing Intergenerational Learning [35–37] and Transformative Learning [38–40].

The second type of IGPs includes programs addressing institutionalized older people with cognitive impairment. These are carried out mainly in nursing homes and day-care centres [41–44]. Both types of IGPs mainly foresee the interaction between elderly people and young adults, such as high school and college students [29–31, 33, 34] or between elderly people and kindergarten and primary school children [45]. IGPs targeting teenagers aged 13-15 are rather rare, as it seems to be quite difficult to capture their interest [25]. Although adolescents are still partially addressed by IGPs, recent studies based on IGPs involving adolescents and nursing home older residents [43] provide evidence of the effectiveness of such approach on the two counterparts [43, 44].

Regardless of the place and age range of the recipients, the literature shows that IGPs can have great effects on individuals of both age groups [7, 46], enhancing the way older people think with regard to younger ones and vice versa [47–50]. They can also have a positive impact at a community level, by enhancing trust, sense of community, and reciprocity between generations [24] and promoting broad social networks [23].

Concerning the effects on frail older people, IGPs can improve their emotional well-being and prevent depression [51]. Moreover, IGPs in residential settings can help residents rediscover the value of their lives [44]. In particular, older people with dementia can be encouraged to engage in activities [41] and can identify and report their feelings better, thanks to the relationships with young people [43].

As far as community-dwelling and active older adults are concerned, IGPs may also enhance “generativity” [32, 52–54], for instance, by means of mentoring and tutoring roles that facilitate the intergenerational transmission of knowledge and values. Moreover, IGPs have been found to be effective in enhancing older people's well-being and self-esteem [50], reducing social exclusion and boosting active ageing [55].

With regard to younger age groups, IGPs may increase their life satisfaction [56, 57], advance their prosocial behaviour [58], and keep them away from delinquency [59]. Moreover, IGPs seem to be effective in increasing youngsters' awareness of older people's needs, by helping them overcome age-related stereotypes more easily [28, 60], and promoting a positive attitude towards the older counterpart [45]. Furthermore, adolescents can improve their perceptions of older adults, gain wisdom from their experiences, and better understand the value of their own lives [44].

Previous IGPs carried out in Italy have never included active older people volunteering in nursing homes as mentors for teenagers and frail older people living in residential care facilities and adolescents as a specific joint target group [61]. Thus, the “*Let's Re-Generate*” study represents the first IGP to do so in this country. Implemented as a pilot project in 2012–2013 in a medium-sized city in Central Italy (Ancona), this study moved from the hypothesis that intergenerational exchange might develop the human potentials and enhance the mental well-being of frail older people living in residential settings, i.e., promoting a meaningful life and the satisfaction of basic human needs for autonomy [62], by starting from the residual capabilities in the emotional, psychological, and relational realms.

This study tested an IGP having two main specific objectives: one for the adolescents and one for the institutionalized older adults. The first objective was to promote a better prevention of intolerance and neglect towards older people, by breaking down age-related stereotypes among adolescents, through the awareness of two different ageing patterns: active older volunteers and disabled older people. The second objective of this study was to promote institutionalized older people's social inclusion, emotional well-being, and relational capabilities, by “breaching” the wall of Italian residential facilities, which are predominantly still based on an age-segregated organizational pattern.

Thus, this study was aimed at creating community spaces and activities in which adolescents, institutionalized older adults, and active older volunteers could meet and interact with each other.

## 2. Materials and Methods

The study was developed by using a research-action methodology [63–65], where the “action” consisted in an IGP whose participants, research methods, and carried out activities are described in the following paragraphs.

### 2.1. Participants

The study involved 25 14-year-old students (18 males and 7 females) and three teachers from a junior secondary school; 16 older residents (mean age: 83) and three social workers of a residential care facility for older people (hosting both a nursing home and a day-care centre); and 16 older volunteers (mean age: 70) from two different volunteers associations (Table 1). All actors were based in Ancona, a city in Central Italy of about 100.000 inhabitants.

Adolescents were chosen as IGP recipients from among peers, because, in this phase of their life, they have to build a new self-image and reconcile the changes induced by rapid biological and psychological growth with the strong social pressures coming from the outside world, e.g., the messages and values coming from the media and the informal groups they belong to [66]. At this stage, teenagers may also experiment new forms of autonomy (e.g., making decisions on school and leisure activities, taking the bus to and from school alone without their parents' and grandparents' help), which may lead to a reduction of intergenerational contacts. Since more frequent contacts with grandparents can boost the adolescents' respect for their grandparents' views [67], in the intergenerational relationship gap that can occur during adolescence, some age-related prejudices can insinuate, which might influence the attitude of the teenagers towards elderly people, and result in indifference or even verbal and physical violence.

The secondary school involved in the IGP was chosen due to its promising track record of interest in the field of intergenerational activities, as reflected in its previous participation in a short IGP aimed at transferring digital competences from students to community-dwelling older people.

Institutionalized older people were chosen as recipients of the IGP because they represent a type of older people among the least investigated in Italy. This IGP residential care facility, hosting totally 42 residents in the nursing home and 20 users in the day-care centre, was selected because many of its residents, though disabled, were still in a sufficiently good cognitive condition and thus able to interact with each other and with youngsters. Although in cognitive good health, many users of the day-care centre suffered from challenging physical and psychological conditions, such as arthritis, ictus-related effects, cardiac and respiratory distress, and anxiety.

The presence of active older people volunteering in nursing homes had the objective of providing youngsters with an example of active ageing and with a support to deal with home residents. The two voluntary associations were selected because they involved older volunteers actively engaged in supporting older people in the city. In this way, they represented a positive example of active ageing, potentially contrasting with and/or integrating the condition of frailty characterizing older residents in the nursing home. The first organization, namely,* Associazione per il Volontariato Socio Sanitario*: AVULSS (Association for Social Health Volunteering), is active in the formal care sector (i.e., hospitals and nursing homes). The second organization,* Associazione per l'invecchiamento attivo*: AUSER (Association for the Promotion of Active Ageing), provides different services to help older persons ageing in their own homes.

The research team was composed of a sociologist, a social worker, and an expert in the psychology of communication. Researchers tried to limit the potential investigators' bias through frequent discussions and constant reflective commentaries with a senior researcher in gerontology. This expert acted as a supervisor and kept an external role, since he did not actively participate in carrying out the activities, but observed their execution by taking notes. To keep the reliability of the study as high as possible, researchers paid constant attention to tracking and reporting processes [68].

### 2.2. Research Methodology

The study adopted a qualitative methodology for data collection and analysis. The interview topic-guide for the youngsters covered five conceptual areas: representation of older people; relationship with grandparents; knowledge of and experience in volunteering; knowledge of intergenerational solidarity; and active ageing. Interviews with older people concerned three main issues: self-representation, representation of young people, and intergenerational relationships. The topic-guide of the interviews with older volunteers focused on four main topics: the condition of both day-care centre and residential older people; intergenerational relationships and solidarity; relationships with older and younger people while volunteering; the concept of active ageing in connection with the voluntary activity.

The data collection took place before (T0), during (T1), and after the implementation phases (T2) of the study (Table 2). In the second wave of the data collection (T1), focus groups with students were replaced by anonymous data collection, because during the first wave (T0) many students had felt uncomfortable expressing their own opinions in front of their classmates.

Finally, ethnographic notes, audio and video materials, the collected participants' impressions and feedbacks on the delivered activities, and the relational interaction experienced by the researchers contributed to data building [69, 70]. Particular attention was paid to the descriptions of relationships that were analyzed through four main parameters: subjects' behaviour, physical touching, responsive facial expressions, and eye contact [71].

The interviews with older people and the focus groups with adolescents were audio-recorded and transcribed verbatim. Textual data were managed with the support of MaxQda11 software and analyzed by using qualitative content analysis [72]. The textual content was divided in chunks, which were coded and associated with macrocategories and subcategories, following a tree-chart. The macrocategories referred to the main items that were included in the interview guide. The subcategories arose directly from the interviewees' thoughts, as reported in Tables 4 and 5.

### 2.3. Action Methodology

An action plan was defined in collaboration with the organizations involved, according to a participatory approach. In this way, each actor could contribute and take responsibility for the contents and the delivery of the actions to be performed. The management of the program followed a circular pattern in which plan, action, survey, and analysis alternated continuously and fed each other, in order to reflect the effects of the actions [73]. The project activities and the entire process were assessed through* ad hoc* tools, to gather the opinions of teachers, social workers, and volunteers. These were used as an analytical support, in order to better understand the research-action context soon after the activities took place. In light of these opinions, actions were evaluated and revised.

All activities were intended as instruments to help the interaction between generations, make people aware of their potentials, and facilitate their emotional involvement. Furthermore, they aimed at creating connections and “co-hold” participants' needs [74], by enhancing the resources available in the given context and fostering interactions [75]. Younger and older participants were asked to engage regularly in activities requiring a high level of interaction [76, 77], i.e., at least once every ten days for two hours during their normal school hours.

The IGP started with two meetings designed to prepare each group for the first contact with the other. Two videotapes introducing the students and the older people living in the facility were prepared with the help of teachers and social workers. These were shown to the counterparts, prior to a first face-to-face meeting.

Considering that the most effective way of building relationships involving different generations is to share activities, spend time together, and enjoy it [78], the proposed program included a series of different activities (Table 3): learning sessions based on manual arts; biographical self-narration; playing games; acting; music and choral activities; self-narration [79]; and life stories [48].

## 3. Results

### 3.1. Impact on the Youngsters

The main objective of this IGP regarding adolescents was to deconstruct the age-related stereotypes, by letting them experience two types of ageing: the disabled and institutionalized older people at risk of social exclusion and the older active volunteers in nursing homes.

Table 4 shows the process of categorization, coding, and analysis of quotations arising from the contents of the interviews with adolescents. It provides some examples of how textual contents were divided in chunks, coded, and associated with macrocategories arising from the topic-guide items and subcategories arising from the contents. Among the topic-guide items, only those dealt with by this article are reported in this table, i.e., representations of the other generation, characteristics of the intergenerational relationship, lessons learnt from the IGP, and suggestions for improving intergenerational relationships. The quotation column shows the opinions of the adolescents at T0 (i.e., before the start of the IGP) and at T1 and/or T2 (i.e., after six months from the IGP beginning and at its conclusion, respectively). The column highlights the impact of the project on the representation and attitudes of the adolescents towards older people in nursing home.

Prior to the research-action (in the following paragraph referred to as IGP), students described the relationship between young and elderly people essentially as a “conflict of interests,” as plastically highlighted by one pupil's remark: “*The elderly are a burden for young people who cannot find work… Italy is a country made of old people, and young people have to pay their pensions…*” (Student, male, T0).

Already after the first six months of joint activities, however, students started slowly to change their opinions on older people and to overcome the stereotypes on ageing they originally had: “*Elderly are often disregarded as they are deemed useless, but those I met are certainly not useless! […] The mass-media take it for granted that young people are better than the elderly in politics but I think this is only a prejudice!”* (Student, female, T1).

At the end of the program, the conflict gave way to friendship, empathy, listening, and mutual understanding: “*Friendship between young and older people is possible and is good […] We can try to be more understanding towards older people and put ourselves in their shoes. We should not exclude them from society but exploit their experience for better facing life problems and thus making them feel useful*” (Student, male, T2).

The IGP stimulated the change in preconceptions about the elderly and helped adolescents reflect on the condition of those in nursing homes, while developing critical thinking. Before the beginning of the program (T0), adolescents thought they could only teach the elders the use of new technologies. During the project implementation, however, the young realised they could get in touch with older people through ways that had the characteristics of solidarity, i.e., by volunteering, listening, and providing physical help.

Thanks to the IGP, some students continued meeting older persons in the care facility: “*I would like to come back to older persons and stay longer with them*” (Student, female, T2). Nevertheless, others needed the support of adults and asked their parents and their teachers to keep in touch with older generations, by creating* ad hoc* opportunities to meet older people more often. A girl suggested that school should* “organize meetings with elderly in nursing homes and activities to do together.”*

Adolescents, although prepared for the first meeting with the elders, experienced a strong emotion when they visited the nursing home residents for the first time; some of them were moved and cried to see frail and inactive older people sitting in a wheelchair. Despite this initial impact, the interaction between students and elders led to a progressive increase in the attitude of the youngsters to speak to the elders and involve them in activities, up to playing and joking with them and exchanging looks of complicity.

Thus, we can say that the interaction with the institutionalized older people led to a change in the attitude of the adolescents, who improved their ability to listen, empathy, and understanding of the elders.

The intergenerational exchanges were boosted by older volunteers, who had a mutual-esteem and trusting relationship with the adolescents. The teenagers admired the altruism, the spirit of service, and the listening attitude of the active older volunteers and looked to them as examples to follow. The adolescents often asked the volunteers for advice on how to interact with the older residents, and they agreed together on the most effective means to involve them in the activities.

### 3.2. Impact on Institutionalized Older People

The main aim of the IGP concerning institutionalized older people was promoting their social inclusion, emotional well-being and relational capabilities, by improving their self-esteem and the representation of themselves and that of adolescents.

Table 5 reports macrocategories and subcategories, codes, and quotations arising from the in-depth interviews with institutionalized older people attending the IGP. This matrix focuses on the elders' representations of adolescents, the intergenerational relationship, the impact of the IGP on the elders' self-representation and mood, and suggestions for improving intergenerational relationships.

At the beginning of the project, older people in both the nursing home and day-care centre had a negative image of themselves, because they felt physically limited: “*I cannot do anything for a young person because I cannot use my hands anymore”* (day-care centre, female, 79, T0). They also had a generally negative perception of youth, as many of them thought young students were “egoists,” “dishonest,” “unable to do craft and manual work,” “lazy,” and not devoted to the family: “*Young people are unable to listen and they think only of themselves*” (day-care centre, male, 79, T0). Given these premises, it was not surprising that, in the first test (T0), all interviewed older adults thought that it was not possible to have a relationship with adolescents and believed that they were unable to do anything for them. A woman said, “*There cannot be a relationship between young and older people, because the first are unable to listen and the second are boring. We believe we are in touch with young people, but they do not want to keep contacts with us*” (day-care centre, female, 82, T0).

To plan the specific IGP activities, the researchers had to start from this context of mistrust and resignation. Nevertheless, after the first 15 events of common activities (which took place around Christmas time), the older people's opinion slowly changed and they started defining students as “lively,” “polite,” “kind,” and “clever.” During the implementation of the program, older participants began thinking that it was possible to have a dialogue between generations and that adolescents might somehow be of help to older people.

At the end of the program, seniors felt that the young were ready to listen and to help them and that there could be a friendship between the young and the old, based on closeness, intimacy, and confidence. As an older participant clearly expressed it, “*There can be friendship between us, because young students may be more open with us than with their parents”* (day-care centre, man, 83, T2). An old lady forgot her pains and reported she was feeling better, even if only for a few hours:* “The days when the students come here, I forget my pains and my problems. Instead, the days that they do not come, like today, I feel all my pains and I have negative thoughts” *(day-care centre, female, age 83, T2). An older woman, who was affected by depressive anxiety and used to walk incessantly around the room, became calmer and stopped walking in order to participate in the workshops and games.

At the end of the program, the feeling of being able to have meaningful relationships with younger pupils started building a significant hindrance to the sense of uselessness that some of the older people felt in the nursing home: “*We old people are useless: we are here to take life as it comes and we have no incentives like in the past. Nevertheless, I learnt that I can talk to a guy about my past. I never thought that a young person might be interested in an old man like me…” *(day-care centre, male, 83, T2).

Some older participants also realized that they were still able to do something useful for adolescents: “*I understood that even we old people can give love to young people, if we have the chance to meet them!” *(day-care centre, female, 79, T2); and also “*I realized that I'm still alive! The students improved my mood!”* (day-care centre, female, 82, T2). It goes without saying that some of the older participants suggested very clearly how intergenerational relationships can be improved in our society: “*The relationship may be bettered through more meetings between old and young people, and through more chances to get together”* (day-care centre, male, age 76, T2).

The observation of the interaction between young students and older residents during the first two meetings showed a certain overall distrust among the latter who, sitting stiffly on their chairs, spoke neither to the adolescents nor to the other residents, but only to the volunteers. Nevertheless, during the program implementation, adolescents and older residents learnt to know each other, interact, and speak. The language of the latter became less formal, the looks more understanding, and the gestures more relaxed and they included searching for a physical contact, such as a handshake or a hug.

### 3.3. Impact on Older Volunteers

Older volunteers facilitated the building of the relationship between young and older persons, by supporting students in speaking with the older care recipients and helping the latter carry out the activities proposed. Most of them reported that they were impressed by the friendly relations born from the interaction between students and older people in the nursing home after the first four months' activities. Older volunteers had a positive opinion of young people in general; they had a good relationship with them and thought that students were interested in the program activities, as reported in the following quotation: “*I love young people: they are the future and the hope despite contradictions and mistakes […]. I had a good relationship with the guys. They asked me a lot about myself and about older people's life and wrote everything down. We can talk to the students about many things, but it's better to see us at work […]. We volunteers have been credible witnesses for the boys and the girls we met” (AVULSS volunteer, male, T2).*

Older volunteers drew a picture of the older residents as people feeling abandoned and isolated, because they usually received very few visits from family members and had very few contacts with the external local community. Moreover, the interaction with other residents was often characterized by conflicts, because these relationships were forced and not chosen.

Volunteers contributed also to a better understanding, including among the researchers, of the time perspective in a nursing home. There is first a time “for waiting”: waiting for a son, a daughter, or a grandchild to come and visit. Then, it becomes the time of disillusionment and frustration: “*The old person in a nursing home is often sad, because the time is marked by the waiting for a relative who will not come”* (AVULSS volunteer, female, T0). Despite this discouraging situation, volunteers found that older people were more reactive after the IGP activities and stressed that: “*… it is fundamental to open these facilities to the town, and to let in people from the outside”* (AUSER volunteer, female).

## 4. Discussion

The above study confirms the potentially positive impact IGPs can have on older and younger cohorts, integrating already existing evidence reported by the literature. Thanks to the joint activities shared together for the “*Let's Re-Generate”* study, the young students and older people participating in the program have been able to gradually overcome and almost fully abandon the age-related stereotypes initially present in both groups' perception [28, 47, 60].

On the one hand, the IGP advanced adolescents' prosocial behavior [58] and drove them to recognize the human, relational, and social value of older people despite their disability [45] and to generate new beliefs on which they could build future choices and actions, for instance, by becoming volunteers themselves [38–40]. In this perspective, the program reached the goal of boosting reciprocity between generations [24] and went beyond it by encouraging adolescents' critical thinking about institutionalized and dependent elderly's living conditions. Many students, indeed, were outraged after the visit to the nursing home and a student, after a lesson reporting data on elder abuse and neglect in Italy, had the courage to report (privately to a researcher) the case of domestic violence perpetrated against an elderly member of his family (which was then reported to the competent authorities). This episode confirms the importance of educating the younger generations on ageing related issues in order to build up a society based on intergenerational solidarity.

On the other hand, the study gave older participants the opportunity to meet younger people and to be part of the community, by reducing social exclusion [50]. In addition, thanks to the IGP, institutionalized elders could reflect on and change the representations of both themselves and their younger counterparts and could reassess their own life pathways [44, 80, 81]. This occurred partly because they could read their past life experiences under a different light, i.e., through the new relationships built with the students contacted via the IGP [62]. Playing and singing together provided a stimulus for cheerfulness that distracted them from their own physical and psychological difficulties, thus improving their own state of mind and psychological and emotional well-being [43, 51].

While the program was expected to impact on older residents' emotional well-being, it was not expected to have an effect on their mental health. This is the case of the older woman affected by depressive anxiety wandering around the room of the day-care centre, who stopped doing so because she was attracted by the activities carried out by the youngsters. The day-care centre social workers reported that they had tried to involve her in various activities many times, without success. It can be argued that the adolescents in the nursing home distracted the lady from her depressive anxiety and persuaded her to sit down, play with teenagers, and interact with them, albeit for a limited time. If we consider that depression is a loneliness correlated disorder quite commonly found among institutionalized older people [82–84] and that experts suggest the adoption of psychosocial interventions for its prevention [17, 85], this unexpected result of the study suggests that psychosocial interventions based on intergenerational relationships can be effective in preventing the onset of depression among residents of nursing homes with fewer emotional ties.

Older volunteers, in turn, had the chance of mentoring young students and this strengthened their generativity, mentoring capabilities [32, 52–54], and active ageing attitude [55]. This finding suggests including older volunteers in future IGPs to train new generations of volunteers and increase the propensity to care for disabled and elderly people. This could have important effects on the formal and informal care sector and also on the welfare state policies [9].

The main novelty of this study lies in implementing an IGP targeting adolescents and institutionalized disabled older people as a joint target group and in additionally involving older volunteers as mentors of the adolescents, helping the latter to more easily understand the living conditions of nursing home residents. Another novelty was bringing the elders out of the residential facility: once to school, twice to the conference room of the city Municipality building, twice to the headquarters of the voluntary associations, and one last time to participate in the final event open to policy makers and citizens. These visits helped older people overcome the wall of isolation from the rest of the community that is partly the consequence of the persistence of age-segregated services and of the lack of economic and human resources for social animation, leisure, and social interaction, especially in Italy [17–20]. This pilot study, therefore, provides important information on the condition of social isolation of the institutionalized elderly people in Italy and, at the same time, identifies in IGPs a possible tool for boosting their inclusion into the community. Despite its significant findings, this study presents some limitations. The first concerns the limited number of participants, which might impinge on the generalizability of its results. As this was a pilot study, the limited number of participants in the IGP was methodologically adequate, and it justified the use of a qualitative methodology. Quantitative data, in fact, would not have had statistical significance. However, the use of quantitative tools, such as psychometric scales, would have made it possible to quantify the impact of the project activities on the cognitive and emotional spheres of the institutionalized older people and on their overall well-being. Thus, the use of only qualitative tools represents the second limit of the study. A replication of the IGP with a larger sample of both elderly people and adolescents may contribute to consolidate and/or integrate the findings presented here, possibly also via a mix-method approach, including a more substantial quantitative data collection. Third, the IGP was relatively short: this one-year program started a process of change in the participants' opinions and attitudes, but a longer experimentation and assessment would be needed to verify whether changes are long-lasting and able to lead to a concrete evolution in behaviours on a longer term. A follow-up, for example, could verify how many adolescents have continued to visit older people in the facility or have started volunteering activities after the end of the program.

A fourth limit of this IGP was not to involve the middle-age generation, i.e., the older people's children and/or the adolescents' parents, because adults might play a crucial role in enhancing the relationship between young and older people, as “gatekeepers of intergenerational exchange” [67].

## 5. Conclusions

The “*Let's Re-Generate”* study suggests that IGPs may contribute significantly to the improvement of institutionalized older people's mental well-being, by helping them (re)discover their own capacity to react to exogenous and only partially controllable factors, such as disability and affective deprivation, and to recognize and acknowledge their own potentialities, such as their ability to listen, to tell, and to welcome others. In this way, this study suggests that even older people with physical and cognitive impairment may be emotionally and psychologically active despite their disability, if properly stimulated through practices based on intergenerational relationships.

This study can certainly contribute to enrich the debate on the quality of life and the quality of care of older people in nursing homes, both in Italy [18–20] and abroad [86, 87]. It does so by suggesting including the number of contacts with the community and the quality and number of intergenerational exchanges into the factors influencing the satisfaction of older nursing home residents and their quality of life perception, beyond the clinical and practical domains and the relationships with the staff [88–90]. The latter are certainly important, but not sufficient to fully understand the condition of the nursing home residents, at least in Italy. In this regard, this study shows that intergenerational practices can be effectively integrated into usual care provision, in ways that can greatly improve institutionalized older people's well-being, by acting on their emotional realm and residual relational capabilities. In light of the above, it might be suggested that IGPs should become a systematic component of care provision in residential facilities for older people. To this purpose, it is key to provide appropriate training opportunities for residential care staff on theoretical and empirical foundations of IGP practices and to provide for adequate resources so that such a strategic tool can become a routine component of elder care in residential contexts.

## Figures and Tables

**Table 1:** Typology of participants.

	*Students*	*Volunteers*	*Users of residential and day-care services*
Males	18	6	5
Females	7	10	11
Total	25	16	16

Mean Age	14	70	83

**Table 2:** Data Collection Tools per Typology of Participants.

*Participants*	*September 2012 (T0)*	*February 2013 (T1)*	*June 2013 (T2)*
Students	Focus Group	Interview	Interview
Older persons	Interview	Interview	Interview
Volunteers	Focus Group	Focus Group	Focus Group

**Table 3:** Description of IGP Activities.

*Time*	*Main objective*	*Specific goals*	*Activities*	*Number*
*September 2012*	*Assessing stereotypes and prejudices*	*Identifying stereotypes*	*Focus groups and interviews*	*2*
October	Overcoming stereotypes between generations	Increasing reciprocal knowledge	Watching videos of students and older people introducing themselves and discussion about them	4

November	Fostering relationship	Introducing younger generations to the dynamics of long-term care	Visit of students to older people in rest home and day care centre	1

December	Fostering relationship	Reinforcing the collaboration between counterparts	Acting class led by an older woman and final show before Christmas	8

	Promoting residual capabilities of older people	Enhance manual skills of elderly	Laboratories for hand made products and exchange of Christmas gifts and visit of older people to adolescents' school	2

January 2016	Assessing stereotypes and prejudices	Identifying stereotypes	Focus groups and interviews	2

February	Fostering dialogue between generations and educating on intergenerational solidarity	Increasing knowledge of active ageing and volunteering with older people	Students and elderly people' visit to headquarters of voluntary associations	2
			Testimonies of volunteer at school	2

March/April	Promoting intergenerational learning	Fostering dialogue	Drawing and comparing the family trees (students and older people)	1
		Fostering collaboration	Preparation of final public event: musical and choral laboratories Older people reached adolescents in a Municipality public building.	6

May	Raising the awareness of citizens and community about intergenerational relationships	Dissemination of project results	Final public event Older people attended the event in a room of the city Municipality	1
	Assessing stereotypes and prejudices	Program assessment	Focus groups and interviews	2

*Total intergenerational meetings*				33

**Table 4:** The process of categorization, coding and analysis of quotations of adolescents attending the IGP at Time 0 and Times 1 and 2.

*Macro-categories from topic-guide items*	*Sub-categories from contents*	*Codes for the analysis*	*Quotations from the interviews*	
			*Time 0*	*Times 1 and 2*
Representation of older people	Physical/cognitive condition	Bad/good	*My grandparents ride a bike and play sudoku*	*Elders are disabled, they are not autonomous because depend on others*
	Behaviour and psychological characteristics		*My grandparents always expect to be right*	*I realized that there are not only grumpy elders people, but also friendly and nice seniors*
			*My grandfather has a very rigid mentality*	*I learnt that we should not misjudge elders*
	Social/economic role	Burden	*The “elderly” are a burden for young people who cannot find work*	
		Experience exploitation	*/*	*We should not exclude older people from society but exploit their experience to better face life problems and thus making them feel useful*
		Usefulness	*/*	*“Elderly” are often disregarded as they are deemed useless, but those I met are certainly not useless*

Intergenerational relationship	Kind of relationship	Conflictual	*Italy is a country made of “elderly”, and young people have to pay their pensions…*	
		Friendly	*/*	*Friendship between young and older people is possible and it is good *
	What young people can do for older people	Teach ICTs	*Young people can teach the technology and the use of social networks*	
		Understanding	*/*	*We can try to be more understanding towards older people, put ourselves in their shoes*
				*We can try not to make fun of older people*
		Listening	*/*	*I understood that even small things like listening can help an old person*
		Psychological support	*/*	*We can sustain older people psychologically*
	What older people can do for young people	Tell	*They can tell their experiences and their mistakes in life*	
		Read	*/*	*They can read books to us*
		Teach and Mentor	*/*	*They can teach us with their experience to face the difficult choices of life*
		Take care	*/*	*They can cook and tell stories*
				*They can understand us and trust us*
				*They can accept us as we are*

Lesson learnt from	The contact with older people in nursing home	Awareness	*Not applicable*	*I understood that unfortunately many elderly people feel neglected by us*
				*An old man was finally moved and this made me realize that we guys did a lot for him*
	Older volunteers' mentoring	Changes of attitudes towards volunteering	*Not applicable*	*I learnt that I can always find space in the day to help someone, even if I am very busy*
				*I would like to volunteer but I would need the help from my parents to do it*
				*I would like to come back to older persons and stay longer with them*

		Appreciation	*Not applicable*	*I do not think I could volunteer with the elderly because I do not have much patience and because sometimes being with the elderly makes me sad. But I really admire people who do it!*

Suggestions for improving the relationship with the youngest	Intergenerational meetings in nursing home	The role of adults	*Not applicable*	*Adults can help the relationship between young and old by taking us from older relatives and making us stay with them a little bit*
		The role of the school authority	*Not applicable*	*The school can incentivize the relationship between us and the elderly by proposing this project to other classes*
				*The school should organize meetings with the eldely in thes in nursing home and activities to do together*

**Table 5:** The process of categorization, coding and analysis of quotations of older people attending the IGP at Time 0 and Times 1 and 2.

*Macro-categories from topic-guide items*	*Sub-categories From contents*	*Codes For the analysis*	*Quotations from the interviews content and associated with the macro and sub-categories*	
			*Time 0*	*Times 1 and 2*
Representation of young people	Negative representation	Egoist	*Young people think just of themselves*	
			*We did love our parents, while young people do not love their parents today*	
	Positive representation	Altruist		*The adolescents I could approach were ready to help, they were not indifferent *
		Able to listen		*They were open to listening the reasons for our problems*

Intergenerational relationship	Kind of relationship	Indifference/distance	*There cannot be a relationship between young and older people, because the first are unable to listen and the second are boring […]they (adolescents) do not want to keep in contact with us*	
		Friendship	*/*	*There can be friendship between us, because young students may be more open with us than with their parents*
	What young people can do for older people	Nothing	*Young people cannot do anything for me: they can just spend money and go dancing*	
		Physical help	*/*	*I can lean on them physically to walk On the practical side, kids can give physical help. *
		ICT literacy	*/*	*They can teach us the computer and their new language.*
		Company	*/*	*A good word and company.*
	What older people can do for young people	Nothing	*I cannot do anything for a young person because I cannot use my hands anymore*	
		Love	*/*	*Even we old people can give love to young people, if we have the chance to meet them!*
		Listening each other	*/*	*I can talk to a guy about my past. I never thought that a young person might be interested in an old man like me*

Impact on	Self-representation	Able to feel emotions	*Not applicable*	*I realized that I'm still alive! *
		Able to give love	*Not applicable*	*Even we old people can give love to young people*
	Mood		*Not applicable*	*The students improved my mood*

Suggestions for improving the relationship with the youngest	Intergenerational meetings in nursing home	More meeting chances	*Not applicable*	*The relationship may be bettered through more meetings between old and young people, and through more chances to get together*
		Talking	*Not applicable*	*The best thing was talking to them. *
		Systematic visits	*Not applicable*	*It would be wonderful if the young people came every day!*
		More time to spend together	*Not applicable*	*Unfortunately the boys have spent too little time with us*
		Trips outside and fun	*Not applicable*	*I would like to take some trips with them out of the nursing home *

## Data Availability

Data related to the results reported in this article are available (in Italian language) at the following link (Study Report and other resources) http://www.inrca.it/INRCA/News2.asp?ID=93.

## References

[1] Bengtson V. L. (2001). Beyond the nuclear family: The increasing importance of multigenerational bonds. *Journal of Marriage and Family*.

[2] Dykstra P. A. (2010). *Intergenerational family relationship in ageing society*.

[3] Coole D., Frost S. (2010). *New materialisms: Ontology, agency, and politics*.

[4] Schadler C. (2016). How to Define Situated and Ever-Transforming Family Configurations? A New Materialist Approach. *Journal of Family Theory and Review*.

[5] Dykstra P. A. (2018). Cross-national Differences in Intergenerational Family Relations: The Influence of Public Policy Arrangements. *Innovation in Aging*.

[6] Drury L., Hutchison P., Abrams D. (2016). Direct and extended intergenerational contact and young people's attitudes towards older adults. *British Journal of Social Psychology*.

[7] Drury L., Abrams D., Swift H. J. (2017). *Making intergenerational connections: What are they, why do they matter and how to make more of them*.

[8] Abrams D., Vauclair C. M., Swift H. (2011). *Predictors of attitudes to age across Europe*.

[9] Albertini M., Kohli M., Vogel C. (2007). Intergenerational transfers of time and money in European families: Common patterns - Different regimes?. *Journal of European Social Policy*.

[10] Eurostat (2016). *Population structure and ageing*.

[11] CENSIS & Human Potential Network Research Foundation (2015). *Italy of the generational tribes*.

[12] Da Roit B. (2007). Changing intergenerational solidarities within families in a mediterranean welfare state: Elderly care in Italy. *Current Sociology*.

[13] Lamura G., Chiatti C., Di Rosa M. (2010). Migrant workers in the long-term care sector: Lessons from Italy. *Health and Ageing*.

[14] Di Rosa M., Melchiorre M. G., Lucchetti M., Lamura G. (2012). The impact of migrant work in the elder care sector: Recent trends and empirical evidence in Italy. *European Journal of Social Work*.

[15] Pasquinelli S., Pasquinelli S., Rusmini G. (2013). Badanti. *Italy. How many, who are and what they do*.

[16] ISTAT (2013). *Residential care facilities and social-health facilities*.

[17] Palese A., Del Favero C., Antonio Zuttion R. (2016). Inactive Residents Living in Nursing Homes and Associated Predictors: Findings From a Regional-Based, Italian Retrospective Study. *Journal of the American Medical Directors Association*.

[18] Censi A. (2000). Per un circolo virtuoso dellautonomia degli anziani. Le cure e lautonomia nelle residenze per anziani [For a virtuous cicle of the autonomy of older people. The care and the autonomy of older people in residential facilities]. *Animazione Sociale*.

[19] Mortari L. (2006). *The practice of taking care*.

[20] Montemurro F., Mancini G., Torre F. (2012). *Indagine sulle Residenze Socio-Assistenziali in Italia [Survey on the Residential Care Facilities in Italy]*.

[21] McCrea J. M., Smith B. T., Newman S. (2014). Social Issues addressed by Intergenerational Programs. *Intergenerational Programs. Past, Present and Future*.

[22] Melchiorre M. G., Penhale B., Lamura G. (2014). Understanding elder abuse in Italy: perception and prevalence, types and risk factors from a review of the literature. *Educational Gerontology*.

[23] MacCallum Dr. J., Palmer Dr. D., Wright Dr. P., Cumming-Potvin Dr. W., Brooker M., Tero C. (2010). Australian perspectives: Community building through intergenerational exchange programs. *Journal of Intergenerational Relationships*.

[24] Jarrott S. E., Morris M. M., Burnett A. J., Stauffer D., Stremmel A. S., Gigliotti C. M. (2011). Creating Community Capacity at a Shared Site Intergenerational Program: "Like a Barefoot Climb Up a Mountain". *Journal of Intergenerational Relationships*.

[25] Melville J. (2016). The Development of an Intergenerational Centre in the UK: How Several Generations Used the Centre and Interacted with(in) the Building. *Studia paedagogica*.

[26] Park A. L. (2015). The Effects of Intergenerational Programmes on Children and Young People. *International Journal of School and Cognitive Psychology*.

[27] Sakurai R., Yasunaga M., Murayama Y. (2016). Long-term effects of an intergenerational program on functional capacity in older adults: Results from a seven-year follow-up of the REPRINTS study. *Archives of Gerontology and Geriatrics*.

[28] Cohen-Mansfield J., Jensen B. (2017). Intergenerational Programs in Schools: Prevalence and Perceptions of Impact. *Journal of Applied Gerontology*.

[29] Roodin P., Brown L. H., Shedlock D. (2013). Intergenerational Service-Learning: A Review of Recent Literature and Directions for the Future. *Gerontology and Geriatrics Education*.

[30] Fair C. D., Delaplane E. (2014). “It is Good to Spend Time with Older Adults. You Can Teach Them, They Can Teach You”: Second Grade Students Reflect on Intergenerational Service Learning. *Early Childhood Education Journal*.

[31] Andreoletti C., Howard J. L. (2018). Bridging the generation gap: benefits young and old. *Gerontology & Geriatric Education*.

[32] June A., Andreoletti C. (2018). Participation in intergenerational service-learning benefits older adults: A brief report. *Gerontology &a geriatrics education*.

[33] Breck B. M., Dennis C. B., Leedahl S. N. (2018). Implementing reverse mentoring to address social isolation among older adults. *Journal of Gerontological Social Work*.

[34] Leedahl S. N., Brasher M. S., Estus E., Breck B. M., Dennis C. B., Clark S. C. (2018). Implementing an interdisciplinary intergenerational program using the Cyber Seniors® reverse mentoring model within higher education. *Gerontoly & Geriatric Education*.

[35] Hoff A., European Commission (2007). Intergenerational Learning as an Adaptation Strategy in Aging Knowledge Societies. *Education, Employment, Europe*.

[36] Hatton-Yeo A., Almeido Pinto T. (2009). Introduction. *Guide for planning and implementing intergenerational projects. Together, yesterday and tomorrow. Mates project*.

[37] Kaplan M., Sanchez M., Hoffman J. (2017). Intergenerational Strategies for Promoting Lifelong Learning and Education. *Intergenerational Pathways to a Sustainable Society*.

[38] Daloz L., J. Mezirow & Associates (2000). Transformative learning for the common good. *Learning as transformation. Critical perspectives on a theory in progress*.

[39] Mezirow J., J. Mezirow & Associates (2000). Learning to think like an adult. Core concepts of transformation theory. *Learning as transformation. Critical perspectives on a theory in progress*.

[40] Mezirow J. (2009). *Transformative learning in practice: Insights from community, workplace and education*.

[41] Lee M. M., Camp C. J., Malone M. L. (2007). Effects of intergenerational Montessori-based activities programming on engagement of nursing home residents with dementia.. *Clinical Interventions in Aging*.

[42] Biggs G. M. J., Knox K. S. (2014). Lessons Learnt from an Intergenerational Volunteer Program: A Case Study of a Shared-Site Model. *Journal of Intergenerational Relationships*.

[43] Baker J. R., Webster L., Lynn N., Rogers J., Belcher J. (2017). Intergenerational Programs May Be Especially Engaging for Aged Care Residents with Cognitive Impairment: Findings from the Avondale Intergenerational Design Challenge. *American Journal of Alzheimer’s Disease & Other Dementias*.

[44] Kim J., Lee J. (2017). Intergenerational program for nursing home residents and adolescents in Korea. *Journal of Gerontological Nursing*.

[45] Senior E., Green J. (2017). Through the Ages: Developing Relationships Between the Young and the Old. *Journal of Intergenerational Relationships*.

[46] Canedo-García A., García-Sánchez J. N., Pacheco-Sanz D. I. (2017). A Systematic Review of the Effectiveness of Intergenerational Programs. *Frontiers in Psychology*.

[47] Weintraub A. P. C., Killian T. S. (2007). Intergenerational programming: Older persons' perceptions of its impact. *Journal of Applied Gerontology*.

[48] Kleyman P. (2000). Life stories: A, nontherapy , for elders and their families. *Aging Today*.

[49] Alcock C. L., Camic P. M., Barker C., Haridi C., Raven R. (2011). Intergenerational practice in the community: A focused ethnographic evaluation. *Journal of Community & Applied Social Psychology*.

[50] Beth Johnson Foundation (2011). *A guide to intergenerational practice*.

[51] Murayama Y., Ohba H., Yasunaga M. (2015). The effect of intergenerational programs on the mental health of elderly adults. *Aging & Mental Health*.

[52] Erikson E. (1982). *The Life Cycle Completed: A Review*.

[53] Baschiera B. (2011). La dimensione formativa e generativa dello scambio intergenerazionale [The formative and generative dimension of intergenerational Exchange]. *Studium Educationis*.

[54] Villar F., Serrat R. (2014). A field in search of concepts: The relevance of generativity to understanding intergenerational relationships. *Journal of Intergenerational Relationships*.

[55] Teater B. (2016). Intergenerational Programs to Promote Active Aging: The Experiences and Perspectives of Older Adults. *Activities, Adaptation & Aging*.

[56] Bostrom A. (2003). *Lifelong learning and social capital. From theory to practice*.

[57] Meshel D. S., McGlynn R. P. (2004). Intergenerational contact, attitudes, and stereotypes of adolescents and older people. *Educational Gerontology*.

[58] Kessler E.-M., Staudinger U. M. (2007). Intergenerational Potential: Effects of Social Interaction Between Older Adults and Adolescents. *Psychology and Aging*.

[59] Whitten T., Vecchio N., Radford K., Fitzgerald J. A. (2017). Intergenerational care as a viable intervention strategy for children at risk of delinquency. *Australian Journal of Social Issues*.

[60] Kaplan M., Wagner J., Larson C. (2001). Child Care/Senior Adult Care Links: Making them Work. *NHSA Dialogue*.

[61] Baschiera B., Deluigi R., Luppi E. (2014). *Intergenerational education. Perspectives, projects and educational methodologies for boosting intergenerational solidarity*.

[62] Ryan R. M., Deci E. L. (2000). Self-determination theory and the facilitation of intrinsic motivation, social development, and well-being. *American Psychologist (Salma)*.

[63] Kemmis S., Mctaggart R. (1982). *The Action Research Planner*.

[64] Cohen L., Manion L., Bell J., Bush T., Fox A., Goodey J., Goolding S. (1984). Action Research. *Conducting Small-Scale Investigations in Educational Management*.

[65] Zanniello G., Scurati C., Zanniello G. (2002). A possible integration between the classic experimentaiton and the research-action. *The research-action, contributions fro the educational development*.

[66] Shaffer D. R., Kipp K. (2014). *Developmental Psychology: Childhood and Adolescence*.

[67] Attar-Schwartz S., Tan J.-P., Buchanan A. (2009). Adolescents' perspectives on relationships with grandparents: The contribution of adolescent, grandparent, and parent-grandparent relationship variables. *Children and Youth Services Review*.

[68] Shenton A. K. (2004). Strategies for ensuring trustworthiness in qualitative research projects. *Education for Information*.

[69] Cardano M., Ricolfi L. (1998). La ricerca etnografica [Ethnographic. *The Qualitative Research*.

[70] Reeves S., Kuper A., Hodges B. D. (2008). Qualitative research methodologies: ethnography. *BMJ*.

[71] Jarrott S. E., Smith C. L., Weintraub A. P. C. (2008). Development of a standardized tool for intergenerational programming: The intergenerational observation scale. *Journal of Intergenerational Relationships*.

[72] Mayring P. (2000). Qualitative content analysis. *Forum: Qualitative Social Research*.

[73] Ebbut D., Burgess G. R. (1985). Educational Action Research: Some general concerns and specific squibbles. *Issues in EducationalResearch: Qualitative Methods*.

[74] Martini E. R., Ripamonti E. (2000). New ways for looking at and thinking elders. *Animazione Sociale*.

[75] Deluigi R. (2010). *Animation for education. How to grow in social engagement*.

[76] Von Rossberg-Gempton I. E., Dickinson J., Poole G. (1999). Creative dance: Potentiality for enhancing social functioning in frail seniors and young children. *The Arts in Psychotherapy*.

[77] Zeldin S., McDaniel A. K., Topitzes D., Calvert M. (2000). *Youth in decision making: A study on the impacts of youth on adults and organizations*.

[78] Bruner J. (1996). *The culture of education*.

[79] Demetrio D. (1996). *Raccontarsi. L’autobiografia come cura di sé*.

[80] Laslett P. (1992). *Una nuova mappa della vita. L’emergere della terza età*.

[81] Saraceno C. (2001). *Età e corso della vita*.

[82] Adams K. B., Sanders S., Auth E. A. (2004). Loneliness and depression in independent living retirement communities: Risk and resilience factors. *Aging & Mental Health*.

[83] Santiago L. M., Mattos I. E. (2014). Depressive symptoms in institutionalized older adults. *Revista de Saúde Pública*.

[84] Jerez-Roig J., de Oliveira N. P. D., de Lima Filho B. F. (2016). Depressive Symptoms and Associated Factors in Institutionalized Elderly. *Experimental Aging Research*.

[85] Minardi H. A., Blanchard M. (2004). Older people with depression: pilot study. *Journal of Advanced Nursing*.

[86] Van Malderen L., Mets T., Gorus E. (2013). Interventions to enhance the Quality of Life of older people in residential long-term care: A systematic review. *Ageing Research Reviews*.

[87] Rodríguez-Martín B., Martínez-Andrés M., Cervera-Monteagudo B., Notario-Pacheco B., Martínez-Vizcaíno V. (2013). Perception of quality of care among residents of public nursing-homes in Spain: A grounded theory study. *BMC Geriatrics*.

[88] Burack O. R., Weiner A. S., Reinhardt J. P., Annunziato R. A. (2012). What matters most to nursing home elders: Quality of life in the nursing home. *Journal of the American Medical Directors Association*.

[89] Mukamel D. B., Harrington C. (2013). Resident satisfaction surveys and clinical quality of care in nursing homes: Two sides of the same coin?. *Aging Health*.

[90] Barsanti S., Walker K., Seghieri C., Rosa A., Wodchis W. P. (2017). Consistency of priorities for quality improvement for nursing homes in Italy and Canada: A comparison of optimization models of resident satisfaction. *Health Policy*.

